# Zinc Chloride Exposure Inhibits Brain Acetylcholine Levels, Produces Neurotoxic Signatures, and Diminishes Memory and Motor Activities in Adult Zebrafish

**DOI:** 10.3390/ijms19103195

**Published:** 2018-10-16

**Authors:** Sreeja Sarasamma, Gilbert Audira, Stevhen Juniardi, Bonifasius Putera Sampurna, Sung-Tzu Liang, Erwei Hao, Yu-Heng Lai, Chung-Der Hsiao

**Affiliations:** 1 Department of Chemistry, Chung Yuan Christian University, Chung-Li 32023, Taiwan; sreejakarthik@hotmail.com (S.S.); gilbertaudira@yahoo.com (G.A.); 2 Department of Bioscience Technology, Chung Yuan Christian University, No. 200, Chung-Pei Rd., Chung-Li 32023, Taiwan; stvn.jun@gmail.com (S.J.); boni_bt123@hotmail.com (B.P.S.); stliang3@gmail.com (S.-T.L.); 3 Guangxi Key Laboratory of Efficacy Study on Chinese Materia Medica, Guangxi University of Chinese Medicine, Nanning 530200, Guangxi, China; 4 Guangxi Collaborative Innovation Center for Research on Functional Ingredients of Agricultural Residues, Guangxi University of Chinese Medicine, Nanning 530200, Guangxi, China; 5 Department of Chemistry, Chinese Culture University, No. 55 Hwa-Kang Rd, Taipei 11114, Taiwan; 6 Center for Biomedical Technology, Chung Yuan Christian University, Chung-Li 32023, Taiwan; 7 Center for Nanotechnology, Chung Yuan Christian University, Chung-Li 32023, Taiwan

**Keywords:** zebrafish, AChE activity, zinc chloride, locomotor behavior, p-Tau, amyloid β

## Abstract

In this study, we evaluated the acute (24, 48, 72, and 96 h) and chronic (21 days) adverse effects induced by low doses (0.1, 0.5, 1, and 1.5 mg/L) of zinc chloride (ZnCl_2_) exposure in adult zebrafish by using behavioral endpoints like three-dimensional (3D) locomotion, passive avoidance, aggression, circadian rhythm, and predator avoidance tests. Also, brain tissues were dissected and subjected to analysis of multiple parameters related to oxidative stress, antioxidant responses, superoxide dismutase (SOD), neurotoxicity, and neurotransmitters. The results showed that ZnCl_2_-exposed fishes displayed decreased locomotor behavior and impaired short-term memory, which caused an Alzheimer’s Disease (AD)-like syndrome. In addition, low concentrations of ZnCl_2_ induced amyloid beta (amyloid β) and phosphorylated Tau (p-Tau) protein levels in brains. In addition, significant induction in oxidative stress indices (reactive oxygen species (ROS) and malondialdehyde (MDA)), reduction in antioxidant defense system (glutathione (GSH), GSH peroxidase (GSH-Px) and SOD) and changes in neurotransmitters were observed at low concentrations of ZnCl_2_. Neurotoxic effects of ZnCl_2_ were observed with significant inhibition of acetylcholine (ACh) activity when the exposure dose was higher than 1 ppm. Furthermore, we found that zinc, metallothionein (MT), and cortisol levels in brain were elevated compared to the control group. A significantly negative correlation was observed between memory and acetylcholinesterase (AChE) activity. In summary, these findings revealed that exposure to ZnCl_2_ affected the behavior profile of zebrafish, and induced neurotoxicity which may be associated with damaged brain areas related to memory. Moreover, our ZnCl_2_-induced zebrafish model may have potential for AD-associated research in the future.

## 1. Introduction

Currently, there are more than 50 million people living with Alzheimer’s Disease (AD) in the world; this is greater than the total population of Spain and is projected to reach nearly 150 million by 2050 [1]. Loss of memory is the first symptom reported by patients suffering from AD. It was hypothesized that the pathogenesis of AD is diverse, including free-radical-mediated processes [2], dysregulated membrane metabolism [3], trace element neurotoxicity [4], or combinations of the abovementioned factors. Another study suggested that the pyruvate transporter on the inner mitochondrial membrane impacts oxidative stress and lipid metabolism, playing a significant role in the pathogenesis of AD [5]. Among these factors, one which gained significant attention is the involvement of trace elements that caused toxicity during AD progression. Previous studies showed that imbalances of trace elements in homeostasis, such as bulk copper [6], bulk iron [7], and zinc [8,9,10], played an important role in the formation of senile plaques in the brain.

Zinc is an essential trace element, required for the functional integrity of many organ systems, as well as for development, growth, and tissue repair [11,12]. The divalent ion forms of zinc are nutritionally essential trace elements for all living organisms, and is also a co-factor for a number of enzymes [13]. However, excessive zinc is lethal and harmful in pathological and toxicological aspects. Zinc was found as an environmental hazard in at least 985 of the 1662 waste sites proposed by the Environmental Protection Agency (EPA) National Priorities list [14]. The toxicity by zinc was reported in several fish species [15,16,17]. It was well documented that zinc induced embryological damage in red sea bream *Pagrus major*, including alterations in fecundity, low hatching rate, and high mortality [18]. Another study revealed that zinc caused toxicity in gills, which altered oxygen consumption, ammonia excretion, osmotic pressure, and the hepatopancreas in several aquatic invertebrates [19].

It was reported that zinc is neurotoxic and causes neurodegeneration in the central nervous system (CNS) [20,21]. Increases in cytosolic Zn^2+^ concentration via transport through Ca^2+^ channels triggered several downstream effects which led to neuronal cell death [22]. Despite the very low levels of free intracellular Zn^2+^, exposure of 400–600 nM Zn^2+^ results in increased toxicity [23]. Moreover, cells with over 400 nM of Zn^2+^ decreased the activity of glycolytic enzymes, such as glyceraldehyde 3-phosphate dehydrogenase (GADPH) and phosphofructokinase [24,25]. Studies also reported that intracellular free Zn^2+^ induced intracellular proton concentration that affected the function of neurons in vitro [26]. Interestingly, a number of studies revealed the significant role of heavy metals in several neurodegenerative diseases, including Alzheimer’s disease, vascular dementia, and Parkinson’s disease [21,27,28].

Reactive oxygen species (ROSs) play an important role in physiological function and are used as a biomarker of oxidative stress in toxicity studies [29,30]. Some studies already showed that the antioxidant defense system and cellular responses to oxidative stress in zebrafish were similar to mammals [31]. In the CNS, the neurotransmitter acetylcholine (ACh), involved in the cholinergic signaling system, is associated with various physiological and behavioral processes, such as motor activity, memory, and cognition, by activating metabotropic muscarinic and ionotropic nicotinic cholinergic receptors [32]. ACh is catalyzed by two cholinesterases: acetylcholinesterase (AChE) and butyrylcholinesterse (BuChE). Zebrafish encodes only the gene for AChE, which is responsible for ACh degradation, and was already identified and functionally detected in the zebrafish brain [33]. Therefore, acetylcholinesterase (AChE) plays an active role in the nervous system [34]. Moreover, AChE is a significant biomarker for detecting environmental contaminants deposited in zebrafish [35,36]. AChE is involved in the termination of impulse exchange through rapid hydrolysis of the neurotransmitter ACh, resulting in enzyme inactivation induced by various inhibitors, and leading to disrupted neurotransmission [37]. As toxic chemicals affect non-target organisms in the aquatic biota, fish are important as bio indicators to monitor the changes in environmental toxicity compared to mammalian models.

The United States Environmental Protection Agency (USEPA) established a system as an initial screen that applied zebrafish as a model for rapid identification of chemical toxicity to evaluate the effects from myriad chemical pollutants [38]. However, the biological effects of ZnCl_2_ on neurobehavioral development in a vertebrate model are not well characterized in research (Table A1). Moreover, some reports found that genotoxic effects and gene expression changes in embryogenesis following ZnCl_2_ exposure were still largely unknown. One recent study demonstrated the toxic effects of zinc in AD in a rat model, proven using histological results [20]. In addition, it was demonstrated developmental effects of ZnCl_2_ during the larval stage of zebrafish [39,40]. Therefore, we sought to clarify both the short-term and long-term effects of ZnCl_2_ on neurotoxicity by investigating the neurological behaviors immediately after exposure and later on by assessing the molecular parameters in adult zebrafish. In addition, there is currently no study on the effect of ZnCl_2_ exposure on memory changes determined using a passive avoidance memory test, and its possible mechanisms in adult zebrafish.

The objective of this study was to determine if exposure to environmentally relevant ZnCl_2_ in adult zebrafish could result in long-term impairment of brain and muscle functions, leading to neurological defects. Therefore, we used different behavioral endpoints to analyze the toxic effects of ZnCl_2_, and assessed the cholinergic system by analyzing ACh and AChE activity in the zebrafish brain. Furthermore, our study was designed to assess parameters related to oxidative stress and the amino-acid neurotransmitter system after long-term exposure to ZnCl_2_. Considering that long-term ZnCl_2_ exposure promotes neurological dysfunction, and the cholinergic system, oxidative imbalance, and antioxidant activity are associated with neurological disorders related to human diseases, different biomarkers including oxidative and biochemical (ROS and malondialdehyde (MDA)), antioxidant parameters (glutathione (GSH), GSH peroxidase (GSH-PX), and superoxide dismutase (SOD)), neurotoxicity (ACh, AChE, amyloid β, and phosphorylated (p)-Tau), neurotransmitters (gamma-aminobutyric acid (GABA), dopamine, and melatonin), and metallothionein protein levels were studied for a better understanding of ZnCl_2_-induced toxicity in the zebrafish model.

## 2. Results

### 2.1. Mortality Curve and Median Lethal Concentration for ZnCl_2_ Exposure

**Figure 1:**
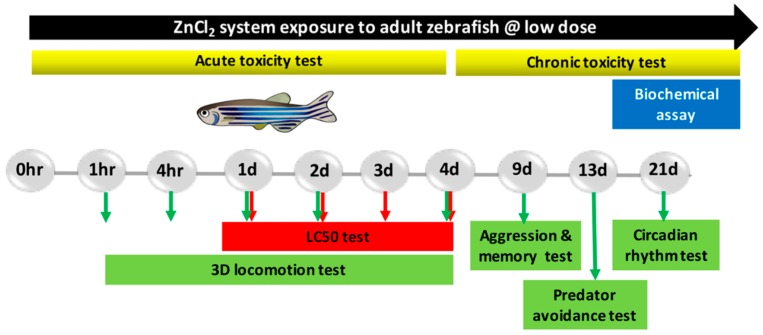
Experimental design scheme of adult zebrafish exposed to ZnCl_2_.

**Figure A1:**
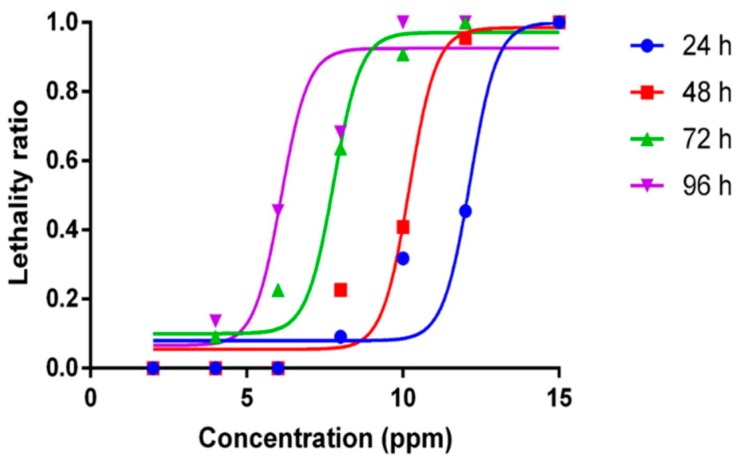
LC_50_ test of ZnCl_2_ in adult zebrafish. Adult zebrafish were incubated with different concentrations of ZnCl_2_ (2, 4, 6, 8, 10, 12, and 15 ppm) and the fish number was recorded every day to build up the mortality curve. LC_50_ curves at 24, 48, 72 and 96 h are labeled in blue, red, green, and purple, respectively.

The acute toxicity test was performed at different concentrations (2, 4, 6, 8, 10, 12, and 15 mg/L) of zinc chloride from low to high concentrations after 24, 48, 72, and 96 h of exposure (Figure 1). No deaths occurred in the untreated control group throughout the exposure experiments. Adult fish incubated at ZnCl_2_ concentrations of 2 mg/L or lower showed mortality similar to the control group, while fish treated with 15 mg/L had 100% mortality after 24 h of incubation. Median lethal concentration (LC_50_) was calculated from the percentage of fish mortality for each ZnCl_2_ concentration with different exposure times (Figure A1). The 96-h LC_50_ of ZnCl_2_ was estimated around 6 ppm.

### 2.2. ZnCl_2_ Exposure Impaired Zebrafish Three-Dimensional Locomotor Activity

**Figure 2:**
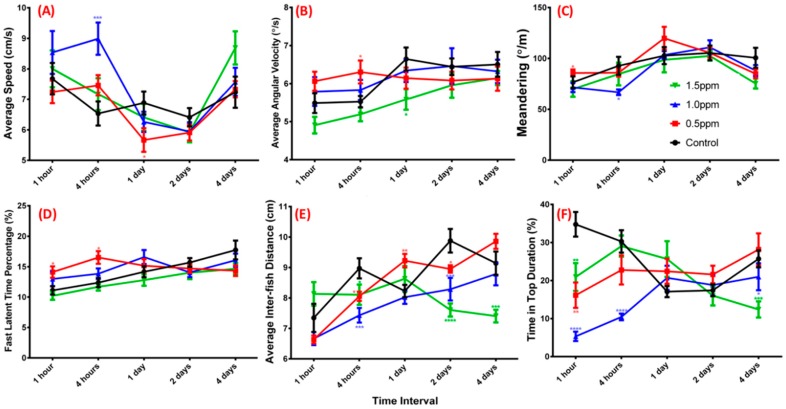
Three-dimensional (3D) locomotor activity assay for ZnCl_2-_treated zebrafish. (**A**) Average speed; (**B**) average angular velocity; (**C**) meandering; (**D**) fast latent time; (**E**) total distance traveled by treated and control groups; (**F**) time spent at top of the tank by adult fish within five different time intervals (*n* = 18 for each testing concentration). Black line, control; red line, 0.5 ppm ZnCl_2_ exposure; blue line, 1.0 ppm ZnCl_2_ exposure; green line, 1.5 ppm ZnCl_2_ exposure_._ Locomotor activity was compared to control at each time point using a *t*-test (* *p* ≤ 0.05; ** *p* ≤ 0.01; *** *p* ≤ 0.001; **** *p* ≤ 0.0001).

The three-dimensional (3D) locomotion behavior of adult fish is a sensitive indicator for testing whether fish are suffering from external stimuli or pollution [39]. We analyzed fish 3D locomotion for five different time periods (1, 4, 24, 48, and 96 h) to demonstrate the acute changes caused by ZnCl_2_ exposure. We observed that exposure to 0.5, 1.0, and 1.5 ppm ZnCl_2_ resulted in a decrease in average speed (Figure 2A, *p* < 0.01), as well as increases in average angular velocity (Figure 2B, *p* < 0.01) and in meandering (Figure 2C, *p* < 0.001). Furthermore, treatment with 1.0 ppm ZnCl_2_ for 24 h increased the time for fast latency with animals showing anxiogenic effects (Figure 2D, *p* < 0.01). The total distances traveled by adult zebrafish treated with ZnCl_2_ were reduced (Figure 2E, *p* < 0.01), and the time spent in the upper zone of the tank by the animals decreased (Figure 2F, *p* < 0.0001) at all exposure concentrations. Generally, when zebrafish are exposed to a new environment, they tend to spend more time at the tank bottom and gradually move to the upper zone after a few minutes. The time spent in the upper zone indicated an anxiolytic-like behavior [40]. Therefore, our results showed that acute ZnCl_2_ exposure induced anxiety-like behavior.

### 2.3. ZnCl_2_ Exposure Reduced Zebrafish Aggression

**Figure 3:**
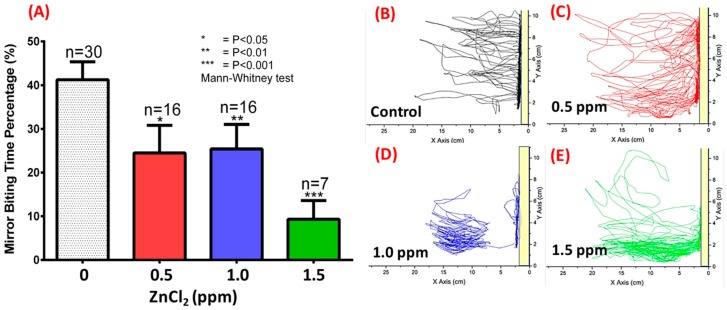
Aggression test monitored by mirror-biting of ZnCl_2_-treated adult zebrafish after nine-day exposure. (**A**) Quantitative comparison of mirror-biting time percentage for zebrafish upon exposure to different concentrations of ZnCl_2_. Statistical analysis was performed using a non-parametric Mann–Whitney test, and compared to control (* *p* ≤ 0.05; ** *p* ≤ 0.01; *** *p* ≤ 0.001). Locomotion trajectories for zebrafish upon exposure to (**B**) 0 ppm (control), (**C**) 0.5 ppm, (**D**) 1.0 ppm, and (**E**) 1.5 ppm ZnCl_2_. The yellow bar indicates the position of the mirror.

We tested fish aggression using the mirror-biting test. Under normal conditions, zebrafish are very aggressive, biting their mirror image [41]. In the untreated control group, we found that adult zebrafish displayed a very high percentage of time visiting/interacting with their mirror images (up to 40%, Figure 3A,B). However, ZnCl_2_ exposure significantly influenced aggressive behavior as determined by the frequency of entries and time spent in the segment near the mirror images (Figure 3B–E). Quantitative data showed that ZnCl_2_-treated animals displayed dose-dependent reductions in aggressive behavior compared to control fish (Figure 3A), and spent more time in the tank bottom as ZnCl_2_ exposure concentration increased (Figure 3D,E; Video S1).

### 2.4. ZnCl_2_ Exposure Reduced Memory in the Passive Avoidance Test

**Figure 4:**
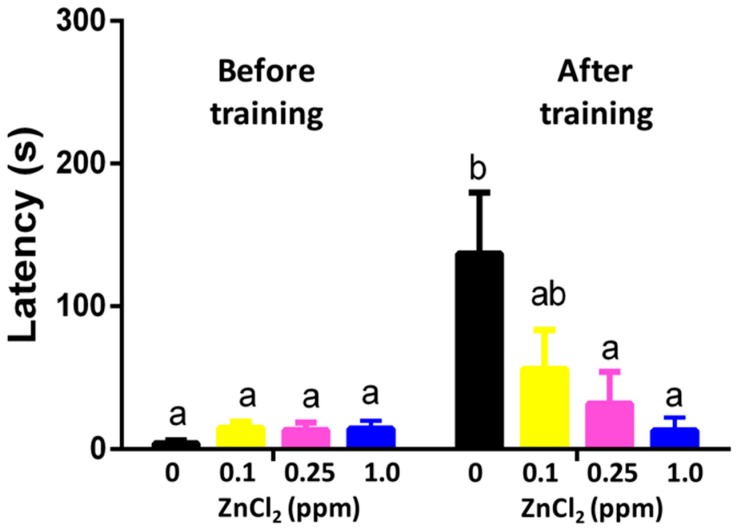
Passive avoidance on training and memorial test after 96 h of exposure to ZnCl_2_ after nine-day exposure. Data are presented as means ± standard error of the mean (SEM) (*n* = 12) and analyzed individually for each treated group. Statistics analysis was performed by one-way ANOVA, followed by Tukey as the post hoc test. Different labels above columns indicate a significant difference with *p* < 0.01 (a, *p* ≤ 0.05; ab, *p* ≤ 0.01; b, *p* ≤ 0.001)).

The adverse effects of zinc on memory were analyzed using the passive avoidance memory test. In the beginning, we acclimated adult zebrafish in the bright chamber after subjecting them to an experimental tank with or without ZnCl_2_. When the separator between the light and dark sides was removed, the untreated fish immediately swam into the dark chamber. Also, we used mild electrical shocks to punish zebrafish to build up the passive memorial connection between the dark side and pain. After being electrically shocked in the dark chamber, zebrafish could remember the pain for several days and displayed longer latency when accessing the dark chamber. We observed that short-term memory was sensitive to ZnCl_2_ exposure, and the reduction in memory retention was dose-dependent. The fish displayed significantly lower memory retention when exposed to very low doses of ZnCl_2_ (0.1 ppm) (Figure 4).

### 2.5. ZnCl_2_ Exposure Does Not Change Predator Avoidance Behavior

**Figure 5:**
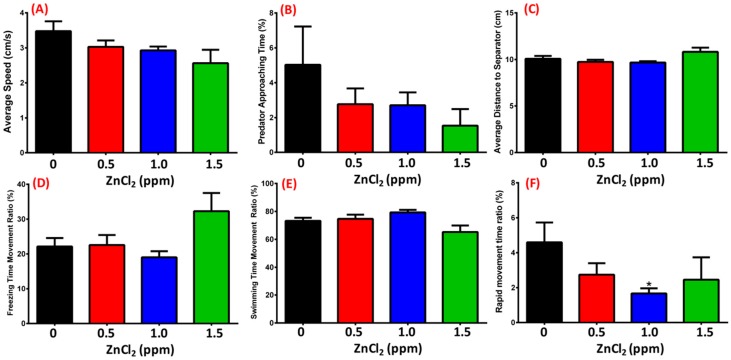
Predator avoidance test after 13-day exposure. (**A**) Average speed; (**B**) predator approaching time; (**C**) average distance of zebrafish to separator; (**D**) freezing movement time ratio; (**E**) swimming time movement ratio; (**F**) rapid movement time ratio of zebrafish. Statistical analysis was performed using a non-parametric Mann–Whitney test (* *p* ≤ 0.05). Sample sizes were 32, 14, 16, and 7 fish for the control, 0.5 ppm, 1.0 ppm, and 1.5 ppm ZnCl_2_-exposed groups, respectively.

Predator avoidance is an innate behavioral response; here, we used the convict cichlid (*Amatitlania nigrofasciata*) as a predator to induce great fear/anxiety. There was no significant change in average speed (Figure 5A), predator approaching time (Figure 5B), average distance to separator (Figure 5C), or freezing/swimming time movement ratio (Figure 5D,E) between control and ZnCl_2_-treated fishes. In contrast, a slightly decrease in in the rapid movement time ratio was observed in the 1 ppm ZnCl_2_-exposed group (Figure 5F, Video S2). This result implied that ZnCl_2_ exposure at our tested concentrations did not change predator avoidance behavior in zebrafish.

### 2.6. ZnCl_2_ Exposure Deregulated the Circadian Rhythm

**Figure 6:**
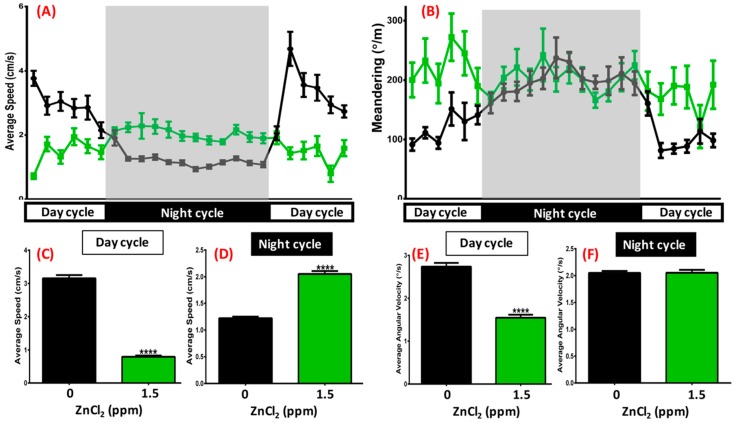
Circadian rhythm test after 21-day exposure. (**A**) Average speed and (**B**) meandering of 1.5 ppm ZnCl_2_-treated (green line) and control fish (black line) during day/night cycle. The gray area shows the dark period and the unshaded area shows the light period. Eighteen fish were observed for both the control and ZnCl_2_-treated groups. Quantitative comparison of average speed during (**C**) day cycle and (**D**) night cycle. Quantitative comparison of average angular velocity during (**E**) day cycle and (**F**) night cycle. Data are presented as means ± SEM (**** *p* ≤ 0.0001).

The light/dark circadian test theoretically depends on the preference of staying in a dark environment, which was validated in behavioral assessment [42,43]. When tracking zebrafish locomotion every hour, we found that untreated fish displayed active locomotion (locomotion speed between 1 and 10 cm/s) during the light cycle and sleeping-like behavior during the dark cycle (locomotion speed less than 1 cm/s) (Figure 6A, black line). However, after 1.5 ppm ZnCl_2_ exposure, zebrafish displayed a reversed circadian rhythm pattern, with less locomotion activity during the light cycle, and higher activity during the dark cycle (Figure 6A, green line). We statistically summarized all locomotion patterns in the light and dark cycles with analysis of average speed during the light cycle (Figure 6C) or dark cycle (Figure 6D). Average angular velocity during the light cycle demonstrated a severe circadian deregulated effect when zebrafish were treated with 1.5 ppm ZnCl_2_ (Figure 6D). Quantitation of the difference in activity revealed that, while control fish decreased their locomotor activity, the ZnCl_2_-treated fish maintained almost a constant level of activity throughout the night.

### 2.7. ZnCl_2_ Exposure Increased Oxidative Stress in the Brain

**Figure 7:**
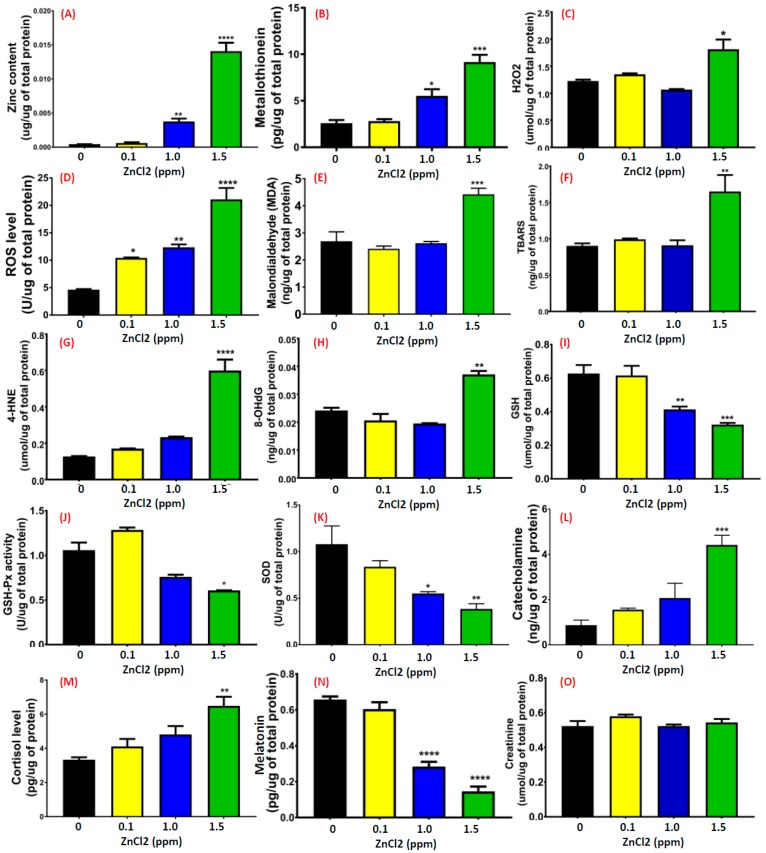
Detection of biochemical parameters in the brain. Enzymatic or ELISA-based methods were applied to detect the levels of (**A**) zinc ions, (**B**) metallothionein, (**C**) reactive oxygen species (ROS), (**D**) H_2_O_2_, (**E**) malondialdehyde (MDA), (**F**) thiobarbituric acid reactive substances (TBARS), (**G**) 4-hydroxynonenal (4-HNE), (**H**) DNA damage marker 8-hydroxy-2′-deoxyguanosine (8-OH-dG), (**I**) glutathione (GSH), (**J**) GSH peroxidase (GSH-Px), (**K**) superoxide dismutase (SOD), (**L**) catecholamine, (**M**) cortisol, (**N**) melatonin, and (**O**) creatinine after treatment with various concentrations of ZnCl_2_. Data are represented as means ± SEM of four independent experiments. Statistical analysis was compared to control using a *t*-test (* *p* ≤ 0.05; ** *p* ≤ 0.01; *** *p* ≤ 0.001; **** *p* ≤ 0.0001).

**Figure A2:**
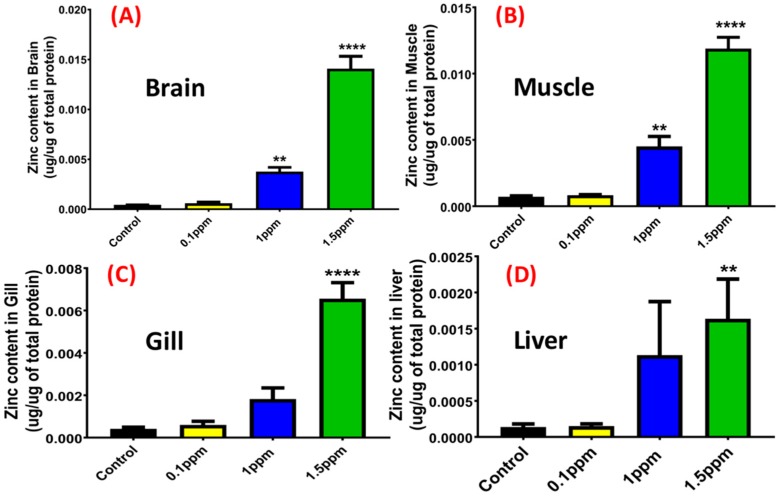
Comparison of zinc levels in different tissues after chronic exposure to ZnCl_2_ at different concentrations for 21 days. Adult zebrafish were incubated with different concentrations of ZnCl_2_, and the relative amounts of zinc accumulation in (**A**) brain, (**B**) muscle, (**C**) gill, and (**D**) liver tissues were measured using enzymatic assays. Data are represented as means ± SEM. Statistical analysis was compared to control using a *t*-test (** *p* ≤ 0.01; **** *p* ≤ 0.0001).

Upon performing a behavioral test, we found that low doses of ZnCl_2_ exposure induced multiple behavioral abnormalities. Next, we attempted to elucidate possible mechanisms by performing biochemical assays. No deaths were found in the treatment group and control groups after acute exposure to ZnCl_2_ at low doses. However, there was a significant increase (more than 30-fold compared to the untreated control) of exogenous zinc ions in the brain after exposure to ZnCl_2_ higher than 1 ppm (Figure 7A). Compared to other tissues, zinc accumulation in the brain showed the highest level (Figure A2). By performing an ELISA assay, we found that the relative amounts of metallothionein (MT, a heavy-metal chelating protein) in the brain and gill tissues also increased significantly (*p* < 0.001) at ZnCl_2_ exposure higher than 1 ppm. In contrast, the MT expression level in the liver tissue was low compared to muscle tissue following 1.5 ppm zinc exposure (*p* < 0.01). A high level of zinc ion accumulation in the brain may induce deteriorative effects; thus, we addressed this question by measuring levels of oxidative species (H_2_O_2_ and ROS), anti-oxidative enzymes (GSH, GSH-Px, and SOD), lipid-peroxidation-related side products (MDA, thiobarbituric acid reactive substances (TBARS), and 4-hydroxynonenal (4-HNE)), DNA damage marker 8-hydroxy-2′-deoxyguanosine (8-OH-dG), and stress hormones (catecholamine and cortisol) in the brain tissue of ZnCl_2_-exposed fish. Results show that oxidative species levels were elevated in the brain (Figure 7C,D). Furthermore, an excessive level of active free radicals induced lipid peroxidation, which was consistent with high levels of MDA (Figure 7E), TBARS (*p* < 0.001, Figure 7F), and 4-HNE (Figure 7G) in the brains of ZnCl_2_-exposed fish. In addition, the persistent high oxidative stress impaired anti-oxidative capacity, which greatly reduced GSH (Figure 7I), GSH-Px (Figure 7J), and SOD (Figure 7K) enzyme activities. By measuring stress hormone levels, we found that brain catecholamine and cortisol levels were significantly elevated fivefold more than the untreated group, which indicated that a low dose of ZnCl_2_ exposure was able to induce significantly high stress in fish (Figure 7L,M). The sleeping-related hormone (melatonin) level was reduced in the brains of ZnCl_2_-exposed fish (Figure 7M), unlike the house-keeping protein, creatinine, which showed a constant level across tissues, providing evidence of the uniformity and consistency of protein extraction and quantification among samples (Figure 7O).

### 2.8. ZnCl_2_ Causes the Downregulation of Neurotransmitter and AD-Related Marker Expression

**Figure 8:**
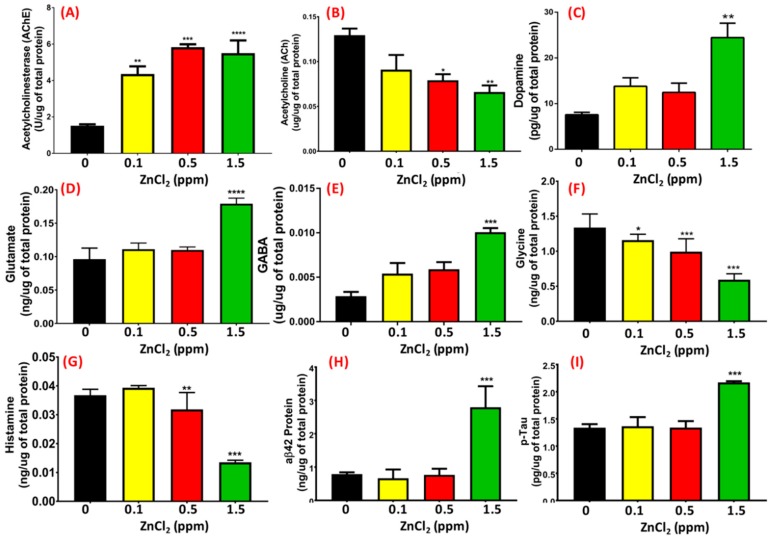
Detection of neurotransmitter levels in the brain. (**A**) Acetylcholinesterase, (**B**) acetylcholine, (**C**) dopamine, (**D**) glutamate, (**E**) gamma-aminobutyric acid (GABA), (**F**) glycine, (**G**) histamine, (**H**) amyloid beta 42 (aβ42), and (**I**) phosphorylated (p)-Tau levels in the brain were measured using ELISA. Data are represented as means ± SEM. Statistical analysis was compared to control using a *t*-test (* *p* ≤ 0.05; ** *p* ≤ 0.01; *** *p* ≤ 0.001; **** *p* ≤ 0.0001).

Behavioral abnormalities, such as lower memory retention, lower aggressiveness, lower locomotion activity, and circadian rhythm dysregulation in low-dose ZnCl_2_-exposed fish led us to ask whether this phenotype was correlated with the downregulation of neurotransmitter and/or other AD-related markers expression. To address this question, we measured the relative content of several important neurotransmitters, such as AChE, ACh, dopamine, GABA, glutamate, glycine, and AD-related proteins like amyloid beta 42 (Aβ42) and p-Tau in the brain using ELISA assays. As shown in Figure 8A,B, AChE activity and ACh levels were detected at various concentrations (0.1, 0.5, 1, 1.5, and 5 ppm) of ZnCl_2_ exposure in zebrafish brain. A significant elevation of AChE activity was detected even at a low dose of ZnCl_2_ exposure at 0.1 ppm (Figure 8A). Interestingly, exposure to 1 and 1.5 ppm ZnCl_2_ resulted in significantly lower ACh levels compared to control, as well as compared to 0.1 and 0.5 ppm exposure (*p* < 0.004, Figure 8B). Carrying out ELISA with antigen-specific antibodies, we found that dopamine (Figure 8C), glutamate (Figure 8D), and GABA (Figure 8E) levels were elevated, while glycine (Figure 8F) and histamine (Figure 8G) levels were reduced. Based on multiple behavioral endpoints measured, we hypothesized that ZnCl_2_-exposed zebrafish display AD-like symptoms, including slow locomotion activity, high anxiety levels, circadian rhythm dysregulation, and lower memory retention. By measuring two AD-related biomarkers in the brain, we confirmed that ZnCl_2_ exposure could significantly elevate the relative contents of Aβ42 (Figure 8H) and p-Tau (Figure 8I).

## 3. Discussion

**Figure 9:**
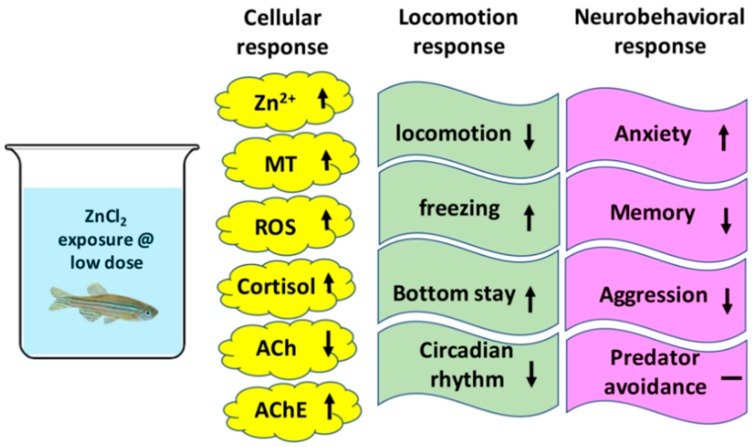
Summary of the toxicological effects of ZnCl_2_ on cellular, locomotion, and neurobehavioral responses in adult zebrafish.

Zinc is widely considered as an essential trace element that is important for human health. In this study, we provided solid evidence showing that chronic ZnCl_2_ exposure can induce behavioral abnormalities and neurochemical changes in adult zebrafish (Figure 9). According to previous studies, the LC_50_ for 96 h of exposure to zinc was found to be 8.46 ppm in *Puntius parrah* and 1.35 ppm in white shrimp [44,45]. In addition, another study suggested that the LC_50_ value of zinc in *Fundulus heteroclitus* was 9.8–75.0 ppm [46]. Our results demonstrated that the 96-h LC_50_ in adult zebrafish was 6 ppm. We hypothesized that minor differences in LC_50_ might be associated with species, developmental stage, test medium, and age. Therefore, the discrepancy observed in LC_50_ was still reasonable.

The zebrafish behavioral model is powerful, efficient, and simple for neuroscience research. Behavioral response was applied for decades as an indicator of toxicological impact within neurons [47,48,49]. Previous studies reported behavioral and locomotor activity alterations influenced by divalent zinc and/or other heavy metals in different species; however, only developmental toxicity of ZnCl_2_ in zebrafish embryos was addressed [50,51,52]. In contrast, our pioneer study demonstrated, for the first time, that adult zebrafish exposed to ZnCl_2_ had reduced locomotor behavior, average speed, entries to the upper zone, and total distance traveled. The average angular velocity and meandering of the fishes increased following 1 ppm ZnCl_2_ treatment compared to the control group, which was consistent with previous studies on other heavy-metal toxicity in zebrafish [53]. According to the literature research, we hypothesize that ZnCl_2_ exposure triggered an increase in muscle cortisol and reactive oxygen species (ROS) levels in the brains of zebrafish, which likely contributed to a stress-elicited reduction in locomotor activity [54].

Previous findings indicated that zinc was one of the potential factors causing senile plaques in the brain regions associated with AD [55]. Zinc at 300 nM showed effects of destabilizing Aβ aggregation and led to fibril formation, which caused damage in the cholinergic system. Our findings demonstrated that low concentrations (0.1 and 1 ppm) of ZnCl_2_ significantly impaired short-term memory in the inhibitory avoidance task, suggesting it caused significant neurotoxic effects. To investigate the mechanism related to the memory impairment induced by ZnCl_2_, we evaluated the activity of biomarkers, acetylcholine esterase (AChE), and acetylcholine (ACh) levels. We found that a low dose of ZnCl_2_ significantly elevated AChE activity and reduced ACh content in the brain tissue of zebrafish. A possible explanation is that ACh reduction may be caused by the excess hydrolysis activity of AChE, thereby altering neuromuscular activity and behavioral response. In addition, our biochemical results showed that levels of the metal-binding protein metallothionein, as well as those of ROS, glutamate, and glycine, were increased, which were associated with neuronal death and caused neurological defects [56]. Therefore, our zinc-induced zebrafish model showed potential as an AD-like model, and may provide a solid platform in neurological research.

Heavy metals are known to affect behavior, including impulsive aggression and anxiety [57,58,59]. In this study, to assess the effects of chronic ZnCl_2_ exposure on social behavior, we performed two behavioral paradigms, the mirror-biting and predator avoidance tests, and observed that the mirror-biting time was significantly reduced by sub-chronic exposure to ZnCl_2_ in adult animals (Figure 6B). Adult zebrafish responded promptly by escape and avoidance, which included freezing, erratic movements, jumping, or shoaling. Nonetheless, predator avoidance was not observed in ZnCl_2_-treated animals sub-chronically in our study; instead, anxiety and fear response toward the predator was expressed in zebrafish with an elevation of cortisol and catecholamine levels in the brain. On the other hand, circadian rhythms can be regulated by circadian genes in the brain or pineal gland in several vertebrates, including zebrafish [60,61]. In our study, the 1.5 ppm ZnCl_2_-exposed fish showed significantly different circadian patterns, with decreased locomotion during the light phase and increased locomotion during the dark phase. Our results showed that GABA expression levels increased and melatonin levels decreased in the brains of fish after exposure to ZnCl_2_. According to a previous study, GABA signals blocked by an antagonist increased circadian rates and rhythm in suprachiasmatic nucleus (SCN) neuron cells [62]. Moreover, disturbance of the pineal melatonin secretion played a significant role in the pathogenesis of Alzheimer’s disease, which was postulated as a circadian disorder [63]. Therefore, we hypothesized that the difference during light/dark phases might be correlated with the inhibition of melatonin in the brains of treated fish, which warrants further investigation in the future.

Previous studies demonstrated that metallothionein (MT) plays an important role in metal detoxification [64] and the homeostasis of zinc and copper [65,66]. Therefore, MTs are considered as a biomarker of environmental pollution of metals, which is consistent with the induction of MTs after chronic exposure to ZnCl_2_ in our study [67,68]. Furthermore, we provided a comprehensive collection of data to show that chronic ZnCl_2_ exposure elevated oxidative stress, as well as lipid oxidation and reactive oxygen species (ROS) levels, which resulted in MT induction.

AD is highly associated with oxidative stress and the overproduction of amyloid Aβ, while ROS levels are significantly higher than seen in healthy normal brains [69]. Over-accumulation of metal ions, inflammation, and aggregation of amyloid Aβs contribute to the high level of ROS in the brain. H_2_O_2_, a major component of ROS, is produced through the Fenton reaction of amyloid Aβ with metal ions and causes the accumulation of inflammatory cytokines such as tumor necrosis factor α (TNFα) and interleukin-1β (IL-1β), which attract active plaques [70]. To investigate the possible mechanism related to memory and other behavioral deficits induced by ZnCl_2_ exposure in zebrafish, we evaluated the impact of ZnCl_2_ on the cholinergic system in the brain. Our results showed that adult animals exposed to ZnCl_2_ had increased activity of neurotransmitters, such as GABA, dopamine, and serotonin, in the brain. Moreover, the inhibition of ACh content in the brain tissue caused behavioral abnormalities. ZnO and Al_2_O_3_ nanoparticles induced DNA damage in human peripheral blood lymphocytes [71]. In this study, single-stranded DNA damage was identified in the brains of ZnCl_2_-treated fish. Our results showed that the brain might be very sensitive to damage at the DNA level following ZnCl_2_ exposure, which impaired memory, decreased locomotion performance, and increased anxiogenic behavior, as well as inducing oxidative stress in brain. Therefore, we suggest that the oxidative stress in the CNS due to zinc exposure may induce changes in social behavior through irreversible DNA damage.

In summary, behavioral and neurotoxicological evaluations of ZnCl_2_ were assessed using multiple biomarker endpoints in our model. The underlying mechanism of ZnCl_2_ toxicity in adult zebrafish resulted from an increase in in oxidative stress biomarker responses (ROS and H_2_O_2_) and a decrease in in antioxidant biomarker responses (SOD, GSH, and GSH-Px). We report novel behavioral and neurotoxic effects upon exposure to divalent zinc in adult zebrafish. Our results demonstrated significant differences in neuroinflammatory effects (TNF α and IL-1β) and single-stranded DNA damage, as well as reductions in locomotion and memory, and alterations in circadian rhythm, in adult zebrafish. Moreover, our study showed that an increase in zinc could serve as a catalyst for increased ROS production, which might be important in the toxicity of amyloid Aβ and p-Tau proteins in brain pathogenesis. These deficits may result in the elevated concentration of MTs and antioxidants within brain tissue, as well as AChE expression in the cholinergic transmission system. Due to the advantage of it being relatively easy to induce an AD-like disease model in adult zebrafish using low-dose ZnCl_2_ exposure, our AD-like neurotoxicological model may benefit future neurodegenerative studies. In addition, it would be worthwhile to unveil the mechanism of Aβ aggregation and Tau pathology using the zinc-induced fish model and applying it to clinical research.

## 4. Materials and Methods

### 4.1. Animal Ethics

All experimental protocols and procedures involving zebrafish were approved by the Committee for Animal Experimentation of the Chung Yuan Christian University (Number: CYCU104024, issue date 21 December 2015). All experiments were performed in accordance with the guidelines for laboratory animals.

### 4.2. Zebrafish Rearing

Wild-type AB strain adult zebrafish (*Danio rerio*) were maintained in a recirculating aquatic system at 28.5 °C with a 10/14-h dark/light cycle according to standards. Circulating water in the aquarium was filtered by reverse osmosis (pH 7.0–7.5). The zebrafish were fed twice a day with lab-grown brine shrimp. For behavioral tests, we used adult zebrafish aged around 6–7 months.

### 4.3. Acute Toxicity Test

Zinc chloride (ZnCl_2_) was purchased from Sigma-Aldrich Corp. (St. Louis, MO, USA). Healthy adult zebrafish were separated into groups of 14 animals aged between six and seven months, and were housed in 3-L tanks prior to treatment. To determine the LC_50_, the following ZnCl_2_ concentrations were used: 2, 4, 6, 8, 10, 12, and 15 mg/L at 24, 48, 72 and 96 h of exposure.

### 4.4. Chronic Toxicity Test

The concentrations of ZnCl_2_ (below 5 mg/L) were chosen according to the USEPA guidelines for the disposal of ZnCl_2_ as an industrial effluent in the environment. Adult fish were exposed to a nominal concentration of 0 (control group), as well as concentrations of 0.1, 0.5, 1, and 1.5 ppm ZnCl_2_ for 24 h, 96 h, and 21 days for acute, sub-chronic, and chronic exposures, respectively. The exposure water was changed daily. The zebrafish were fasted during the entire experiment’s duration. A series of behavioral tests (3D locomotion, predator avoidance, memory, aggression, and circadian rhythm) were conducted in all groups, and performance was compared between ZnCl_2_-treated and control groups (procedures are summarized in Figure 1).

### 4.5. Adult Neurobehavioral Analysis

Five behavioral assays were used to assess neuron function in adult zebrafish upon systematical exposure to low concentrations of ZnCl_2_. The test battery consisted of 3D locomotor activity (for acute toxicity test), passive avoidance (on day 9 of ZnCl_2_ exposure), aggression (on day 9 of exposure), predator escape/avoidance (on day 13 of exposure), and circadian rhythm (on day 21 of exposure) assays which are described below.

### 4.6. Zebrafish 3D Locomotor Activity

Three-dimensional locomotor activity was tracked at five separate time points after exposure to ZnCl_2_ for 1, 4, 24, 48, and 96 h. The tracking strategy for zebrafish 3D locomotion followed the protocol described in our previous study [39]. In each test, we routinely tested the 3D locomotion activity of six fish at each time point while holding them in an acrylic tank (20 × 20 × 20 cm with a 15-cm-high water level). We tested in triplicate and had a total number of 18 fish for both the untreated and ZnCl_2_-treated groups.

### 4.7. Passive Avoidance Test (Short-Term Memory Test)

To evaluate the effects of ZnCl_2_ on short-term memory impairment in zebrafish, the passive avoidance test was carried out after 96 h of exposure, following a previous protocol with minor modification [72]. The test included two sessions with an interval of 24 h between sessions. In each session, fish were placed individually in an acrylic tank (30 × 20 × 20 cm) divided by a door into black and white compartments with equal size. In the training session, the fish were placed in the white compartment with the door closed for one minute for environmental acclimatization and recognition. Next, the door between the compartments was lifted and the fish were allowed to cross over to the dark side of the tank. When fish passed into the dark compartment the door was again closed, and a mild electric shock (25 V, 1 mA) was subjected to the fish with a 5-s interval. When fish passed into the white compartment, fish were pushed back into the dark compartment and shocked again until the fish returned into the white compartment. The training was repeated for a maximum of three times or until the fish passed to the white side. The fish that completed the training were stored in a small plastic container (15 × 10 × 5 cm) and kept at 28 °C. After 24 h, the fish were put into the training apparatus and tested for latency to pass into the dark area. The latency observed in the test session was used as an indicator of short-term memory retention.

### 4.8. Aggressiveness Test

Aggressive behavior was determined in adult zebrafish based on the mirror test as previously described with minor modification [73,74]. The test tank (28 × 5 × 15 cm) was filled with 1 L of water where a mirror was located at the side of the tank. One minute after the fish were introduced into the tank, aggressive behaviors (average speed, maximum and minimum speed, distance traveled, freezing, swimming, and rapid time movement percentage, mirror-biting time percentage, and longest duration in mirror side percentage) were recorded for a period of 5 min.

### 4.9. Predator Avoidance Test

To analyze fear-like and escaping behavior in zebrafish, the predator avoidance test was performed based on the mirror test described previously with minor modification [75]. The test tank (28 × 5 × 15 cm) was filled with 1 L of water where a transparent separator was in the middle of the tank. The predator convict cichlid (*Amatitlania nigrofasciata*) was put into the left area and zebrafish were put into the right area. After the fish were introduced into the tank, the predator avoidance behaviors (average speed, maximum and minimum speed, distance traveled, freezing, swimming, and rapid time movement percentage, top/bottom ratio of time spent and traveling distance, predator approaching time percentage, and distance to predator separator on average) were recorded for a period of 5 min.

### 4.10. Circadian Rhythm Test

To analyze zebrafish locomotion activity during the day and night, we performed a circadian rhythm test based on a previously published method with some modifications [76]. To prevent any external disturbance, the tested fish were moved into a temperature-controlled incubator and kept at 28 °C. One specially designed light-emitting diode (LED) light box with conventional and infrared LED arrays was used as the bottom light source. One infrared-sensitive charge-coupled device (CCD; detection window: 700–1000 nm) with a maximum resolution of 1920 × 1080 pixels and a 30-fps frame rate was used for video recording (3206_1080P module, Shenzhen, China). In this experiment, we recorded zebrafish locomotion activity (average speed, average angular velocity, and meandering) for 1 min every hour and used the idTracker software to track fish movement trajectories according to our previously published method [39].

### 4.11. Biochemical Parameters

#### 4.11.1. Tissue Preparation and Total Protein Determination

After performance of the behavioral experiments, fish were euthanized with a high concentration of 200 mg/L tricaine solution (MS-222). For each independent assay, the whole brain and tissue from the caudal peduncle were extracted. A pool of three zebrafish tissues was used to prepare an independent homogenate preparation for each sample, which was homogenized on ice in 50 volumes (*v*/*w*) of phosphate-buffered saline (PBS) at pH 7.2. Samples were then centrifuged at 15,000 rpm for 15 min at 4 °C, and the supernatant was kept in microtubes at −80 °C for further assays. Total protein concentration was determined using a Pierce BCA Protein Assay Kit (23225, Thermo Fisher Scientific, Waltham, MA, USA). The color formation was analyzed at 562 nm using a microplate reader (Multiskan GO, Thermo Fisher Scientific, Waltham, MA, USA).

#### 4.11.2. Analysis of Zinc and Metallothionein (MT) Content in Tissues

Concentrations of zinc in the brain and other tissues were determined using a zinc calorimetric quantification kit (E011, Nanjing Jiancheng Bio-Engineering Institute Co., Ltd, Nanjing, China). Tissue samples were extracted and prepared using PBS according to the method described in the above section. Calibration curves were prepared using serial dilutions of stock solutions of 100 mg/L zinc standard. The color formation was analyzed at 630 nm using a microplate reader (Multiskan GO, Thermo Fisher Scientific, Waltham, MA, USA). A fish metallothionein (MT) ELISA kit was used to measure total MT in tissues according to the manufacturer’s instructions (ZG-E1562, Zgenebio Company, Taipei, Taiwan). The color change was measured spectrophotometrically at a wavelength of 450 nm. MT content was expressed as pg/mL.

#### 4.11.3. Determination of Acetylcholine and Acetylcholinesterase in Brain Samples

ZnCl_2_-treated zebrafish were anesthetized and euthanized using 200 mg/L tricaine. The whole-brain tissue was homogenized on ice in 50 volumes (*v*/*w*) of PBS at pH 7.2 using a bullet blender tissue homogenizer (Next Advance, Inc., Troy, NY, USA). For the AChE activity analysis, a pool of brain tissue from three individual fish was considered as one sample. The tests were performed in triplicate using a total of nine fish per group to ensure consistency. The activity of the homogenate was measured using an AChE calorimetric kit (A024, Nanjing Jiancheng Bio-Engineering Institute Co., Ltd., Nanjing, China) in accordance with the instructions. Briefly, 10 μL of brain homogenate was placed onto the plate. Then, 100 μL of working solution containing AChE enzyme, assay buffer, dye reagent, and enzyme mix was added into each well. After incubation for 15 min, the color formation was analyzed at 412 nm using a microplate reader (Multiskan GO, Thermo Fisher Scientific, Waltham, MA, USA) and the AChE activity was expressed in μmol. ACh levels were determined using an ACh ELISA kit (ZG-E1585, Zgenebio Company, Taipei, Taiwan) according to the specifications of the manufacturer. The absorbance was analyzed at 450 nm using a microplate reader (Multiskan GO, Thermo Fisher Scientific, Waltham, MA, USA). Data were expressed as U/mg of total protein.

#### 4.11.4. Quantification of Hydrogen Peroxide and Reactive Oxygen Species Levels in Brain Samples

Hydrogen peroxide (H_2_O_2_) (ZG-E1602, Zgenebio Company, Taipei, Taiwan) and the reactive oxygen species (ROS) ELISA kit (ZG-E1561, Zgenebio Company, Taipei, Taiwan) were applied to detect their respective components according to the manufacturer’s instructions, and absorbance was determined at 450 nm. These assay kits are based on the sandwich ELISA method that involves a specific antibody for the detection of the chemicals of interest.

#### 4.11.5. Determination of MDA, TBARS, 4-HNE, 8-OH-dG, Catecholamine, and Cortisol Levels in Brain Samples

Under oxidative stress, levels of biomarkers for lipid peroxidation (malondialdehyde, TBARS, and 4-HNE), DNA damage marker 8-OH-dG, and stress hormones catecholamine and cortisol were measured in the brain homogenate samples. The production of MDA, TBARS, 4-HNE, and 8-OH-dG in the samples was measured using a commercial ELISA kit (ZG-E1592, ZG-E1605, ZG-E1603 and ZG-E1564, Zgenebio Company, Taipei, Taiwan) according to the manufacturer’s protocol. The levels of catecholamine (CA) and cortisol in the brain homogenate were determined using an ELISA kit (ZG-E1590 and ZG-E1575, Zgenebio Company, Taipei, Taiwan) according to the manufacturer’s protocol. The color change was measured spectrophotometrically at a wavelength of 450 nm.

#### 4.11.6. Determination of Antioxidant Capacity in Brain Samples

The exposure of fish to ZnCl_2_ promotes the production of glutathione (GSH), glutathione peroxidase (GSH-Px), and superoxide dismutase (SOD) in the fish brain, and the relative amounts of each were measured using specific ELISA kits (ZG-E1622, ZG-E1601, ZG-E1604, Zgenebio Company, Taipei, Taiwan). The color change was measured spectrophotometrically at a wavelength of 450 nm.

#### 4.11.7. Evaluation of Neurotransmitter Levels in Brain Samples

Levels of neurotransmitters (GABA, glutamate, glycine, dopamine, 5-hydroxytryptamine (5-HT), norepinephrine, histamine and melatonin) in the fish brain were determined using commercially available ELISA kits (ZG-E1574, ZG-E1588, ZG-E1587, ZG-E1753, ZG-E1572, ZG-E1571, ZG-E1586, ZG-E1597, Zgenebio Company, Taipei, Taiwan) according to the manufacturer’s instructions. The color change was measured spectrophotometrically at a wavelength of 450 nm.

#### 4.11.8. Estimation of Amyloid Beta (Aβ-42) and p-Tau Levels in the Brain

Aβ42 and p-Tau are two markers for Alzheimer’s Disease, and their concentrations in the brain tissues were detected using ELISA kits (ZG-E1607 and ZG-E1618, Zgenebio Company, Taipei, Taiwan) according to the manufacturer’s instructions. The absorbance was analyzed at 450 nm using a microplate reader (Multiskan GO, Thermo Fisher Scientific).

#### 4.11.9. Statistical Analysis

For statistical analysis, the experimental values were compared between groups and data were expressed as means ± standard error of the mean (SEM). The Prism software (Graph pad Software version 7, La Jolla, CA, USA) was used to determine significant differences between treated and control groups. Data were compared using the *t*-test, ANOVA, or non-parametric test, depending on data normality for significance determination. A significant difference was considered to be *p* < 0.001.

## Supplementary Materials

The following are available online at http://www.mdpi.com/1422-0067/19/10/3195/s1, Video S1: Mirror-biting assay of adult zebrafish after exposure to different concentrations of ZnCl_2_. Video is sped up 10×; Video S2: Predator avoidance test of adult zebrafish after exposure to different concentrations of ZnCl_2_. Video is sped up 10×; Video S3: Circadian rhythm test of adult zebrafish after exposure to 1.5 ppm ZnCl_2_. Video is sped up 5×.

## Appendix A

**Table A1:** Comparison of present study with other animal models exposed to ZnCl_2_.

Toxicant	Model Organism	Concentration	Study	Behavior Endpoints	Toxic Effects	LC_50_	References
ZnCl_2_	Mice	7.5, 10, 15 mg/kg	In vivo	No	Cytotoxicity, head abnormalities	NA	[77]
ZnCl_2_	Neuronal cells	0.15 mmol/L	In vitro	No	Loss of viability	NA	[78]
ZnCl_2_ + ZnO	MDKC cells	100 μg/mL	In vitro	No	Cytotoxicity, genotoxicity	NA	[79]
ZnCl_2_	Mice	10 ppm	In vivo	Yes	Depressive Behavior	NA	[80]
Zinc	Mice	equimolar	In vitro	No	Stimulate hippocampal microglia activation	NA	[20]
ZnCl_2_	*D. magna*	0.16 ppm	In vivo	No	Toxic effects on growth and reproduction	NA	[81]
ZnCl_2_ + ZnO	*C. elegans* larvae	614 μM	In vivo	No	Genotoxicity, Apoptosis	NA	[82]
Zinc	Red sea bream	2.5 ppm	In vivo	No	Toxic effects on development and growth	4.3 ppm	[18]
ZnCl_2_	Zebrafish embryo	0.5 ppm	In vivo	No	Skeletal anomalies	NA	[83]
ZnCl_2_	Zebrafish embryo	0.1–0.5 ppm	In vivo	No	Developmental deformities	NA	[84]
This study Low concentration of ZnCl_2_	Adult zebrafish	0.1–1.5 ppm	In vivo	Yes, we used 5 behavioralparameters	Memory impairment, locomotor deficit, and high AChE content in brain	6 ppm	This study
